# A Literature Review and Meta-Analysis on the Potential Use of miR-150 as a Novel Biomarker in the Detection and Progression of Multiple Sclerosis

**DOI:** 10.3390/jpm14080815

**Published:** 2024-07-31

**Authors:** Vasile Calin Arcas, Anca Maria Fratila, Doru Florian Cornel Moga, Iulian Roman-Filip, Ana-Maria Cristina Arcas, Corina Roman-Filip, Mihai Sava

**Affiliations:** 1 Doctoral School, Faculty of Medicine, Lucian Blaga University of Sibiu, 550169 Sibiu, Romania; calin.arcas@ulbsibiu.ro; 2 Faculty of Medicine, Lucian Blaga University of Sibiu, 550169 Sibiu, Romania; corina.roman@ulbsibiu.ro (C.R.-F.); mihai.sava@ulbsibiu.ro (M.S.); 3 Military Clinical Emergency Hospital of Sibiu, 550024 Sibiu, Romania; 4 Department of Neurology, “George Emil Palade” University of Medicine, Pharmacy, Sciences and Technology, 540136 Targu Mures, Romania; roman-filip.iulian.22@stud.umfst.ro; 5 Faculty of Medicine, Iuliu Hatieganu University of Medicine and Pharmacy of Cluj-Napoca, 400012 Cluj-Napoca, Romania; arcas.anamariacristina@elearn.umfcluj.ro; 6 Emergency County Clinical Hospital Sibiu, 550245 Sibiu, Romania

**Keywords:** miRNAs, miRNA-150, miR-150, multiple sclerosis, biomarker

## Abstract

Background: MicroRNA-150 (miR-150) plays a critical role in immune regulation and has been implicated in autoimmune diseases like Multiple Sclerosis (MS). This review aims to evaluate miR-150’s potential as a biomarker for MS, necessitating this review to consolidate current evidence and highlight miR-150’s utility in improving diagnostic accuracy and monitoring disease progression. Methods: A comprehensive literature search was conducted in databases like PubMed, Scopus, Google Scholar, SciSpace, MDPI and Web of Science, adhering to PRISMA guidelines. Studies focusing on miR-150 implications in MS were included. Data extraction was conducted, while quality assessment was done using the NOS and AMSTAR 2 tools. With the extracted data a statistical analyses conducted. Results: 10 eligible articles were included in review. Findings show that miR-150 levels were consistently deregulated in MS patients compared to healthy controls, correlating with disease severity and clinical parameters such as (EDSS) scores and disease activity. Additionally, miR-150 is implicated in the inflammatory pathogenesis of MS, affecting immune cell regulation and inflammatory pathways. Conclusions: MiR-150 is a promising biomarker for MS, showing significant potential for improving diagnostic accuracy and monitoring disease progression. Its consistent deregulation in MS patients and correlation with clinical parameters underscore its clinical utility. Further research should validate miR-150’s salivary presence and its possible usage as a novel biomarker and therapeutic potential in the development of MS.

## 1. Introduction

MicroRNAs (miRNAs) are small [1], noncoding RNAs [2], 18–25 nucleotides [1,3], that regulate gene expression post-transcriptionally and play significant roles in immune function and disease processes [1,2,3,4]. By binding to target mRNAs, miRNAs can either degrade the mRNA or inhibit its translation [5], thereby controlling protein synthesis [6]. In the immune system, miRNAs influence the development and function of various immune cells, including T cells and B cells [7]. Specific miRNAs, such as miR-141 [8], miR-200a [8,9], miR-150 [7,10], miR-223 and miR-326 [11], have been identified as dysregulated in neurodegenerative and demyelinating diseases, influencing the balance between pro-inflammatory and anti-inflammatory processes [8,9,10,11].

One miRNA of particular interest is miR-150 [12], which is highly expressed in lymph nodes, spleen, and the thymus [13]. It regulates the maturation and differentiation of T and B cells by targeting c-Myb, a transcription factor critical for the development of these cells [14]. In autoimmune diseases like Systemic Lupus Erythematosus (SLE) and Rheumatoid arthritis (RA), miR-150 has been found to play a significant role [15], as well as in the modulation of other immune-related pathways, further demonstrating its broad impact on immune regulation across various autoimmune conditions [16]. miR-150 is associated with multiple sclerosis (MS) through its involvement in regulating the maturation and differentiation of immune cells [17], contributing to the inflammatory processes characteristic of the disease [18].

MS is a chronic autoimmune disease that affects the Central Nervous System (CNS) [19], leading to the progressive deterioration of neurological function [20]. It is characterized by an autoimmune response wherein the immune system targets and degrades the myelin sheath that insulates nerve fibers, resulting in impaired signal transmission between the CNS and peripheral tissues. [21]. Symptoms vary widely among patients but commonly include fatigue, difficulty walking, numbness or weakness in limbs, vision problems, and cognitive impairment [22].

MS predominantly affects young adults [23], with most diagnoses occurring between the ages of 20 and 40 [24]. It is more common in women than in men, with a female-to-male ratio of approximately 2:1 [25]. The prevalence of MS also varies geographically, being higher in regions further from the equator [26]. This disease significantly impacts the quality of life and can lead to disability [27], although the course of MS can be highly variable, with some individuals experiencing periods of remission and others facing a steady progression of symptoms [28].

At the immunological level, MS is primarily driven by the dysregulation of CD4+ T cells [29], particularly the Th1 and Th17 subtypes, which become autoreactive and cross the blood-brain barrier (BBB) [30]. Once in the CNS, these cells recognize myelin antigens as foreign, initiating an inflammatory cascade. This immune response involves the activation of other immune cells, including CD8+ T cells, B cells, and macrophages, which further amplify the inflammatory process [31]. B cells contribute to the pathogenesis by producing autoantibodies against myelin components and presenting antigens to T cells, while macrophages and microglia release pro-inflammatory cytokines and reactive oxygen species that exacerbate tissue damage [32].

The chronic inflammation and immune-mediated damage lead to demyelination, axonal injury, and neurodegeneration. Demyelination disrupts the efficient transmission of electrical impulses along nerve fibers [33], resulting in the clinical manifestations of MS, such as motor and sensory deficits, vision problems, and cognitive impairments [34]. In the early stages of the disease, remyelination can occur, leading to partial recovery of function. However, as the disease progresses, the ability of the CNS to repair myelin diminishes, leading to irreversible axonal loss and the progressive accumulation of neurological disability [35]. The development of MS is thus a result of a multifactorial process involving genetic predisposition, environmental influences, and a complex immune response that ultimately leads to CNS damage and functional impairment.

MicroRNA-150 (miR-150) has been identified as a key regulator in the differentiation and function of these T cells [29]. miR-150 modulates the maturation and activation of both CD4+ [29,30] and CD8+ T [36] cells by targeting specific mRNA transcripts that encode proteins critical for T cell development and response [37]. For instance, miR-150 targets the transcription factor c-Myb, which is essential for the proper development of T and B cells [30]. By fine-tuning the expression of c-Myb, miR-150 influences the proliferation and differentiation of these immune cells, thereby impacting the overall immune response. By modulating c-Myb levels, miR-150 indirectly affects the production of cytokines such as IL-17, IFN-γ, and TNF-α [38].

**Figure 1:**
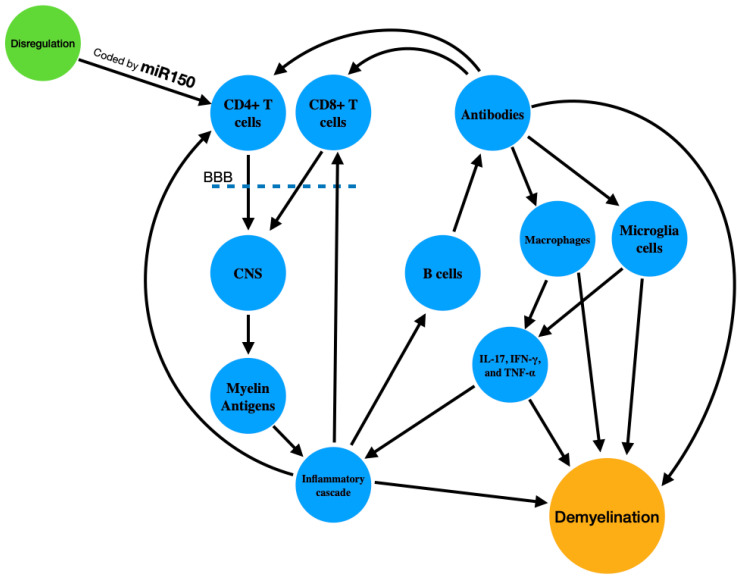
Immunological Mechanism of MS (CD4+ T Cells: Cluster of Differentiation 4 Positive T Cells, BBB: Blood-Brain Barrier, CNS: Central Nervous System, CD8+ T Cells: Cluster of Differentiation 8 Positive T Cells, miR-150: MicroRNA-150, IL-17: Interleukin 17, IFN-γ: Interferon Gamma, TNF-α: Tumor Necrosis Factor Alpha).

CD4+ and CD8+ T cells play crucial roles in the immune system [37], with CD4+ T cells primarily functioning as helper cells that coordinate the immune response [39], while CD8+ T cells act as cytotoxic cells that directly kill infected or aberrant cells. MicroRNA-150 (miR-150) has been identified as a key regulator in the differentiation and function of these T cells [40]. The process in wich the dysregulation of T cells is leading to demyelination is shown is Figure 1. 

MicroRNA-150 (miR-150) has emerged as a promising candidate biomarker for MS [41] due to its significant role in regulating immune responses and its differential expression in MS patients [37,42]. In addition to its diagnostic potential, miR-150 could be valuable in predicting disease progression and treatment responses [43]. Studies have shown that higher miR-150 levels in patients with clinically isolated syndrome (CIS) are associated with a higher likelihood of converting to MS, indicating its potential in early diagnosis [42,43,44]. Moreover, miR-150 levels have been found to decrease in the CSF after treatment with disease-modifying drugs like natalizumab, while plasma levels of miR-150 increase, reflecting changes in immune cell dynamics. This responsiveness to treatment further underscores miR-150 [45].

The aim of this literature review is to systematically evaluate the presence and quantification of miR-150 in saliva and its potential to serve as a novel, non-invasive biomarker for the early detection, diagnosis, and monitoring of MS. This review seeks to explore the biological mechanisms of miR-150 within the immune system, assess its correlation with MS disease activity in various biofluids, and address the feasibility and clinical implications of using salivary miR-150 as a diagnostic tool.

## 2. Material and Methods 

### 2.1. Literature Search Strategy 

To ensure a comprehensive and thorough review, an extensive literature search was conducted across multiple electronic databases, including PubMed, Scopus, Google Scholar, SciSpace, MDPI and Web of Science. The search aimed to identify relevant studies, encompassing a broad range of research on miR-150 as a potential biomarker in MS. The selection of databases was strategic, covering a wide array of biomedical literature, clinical trials, systematic reviews, and relevant scientific articles. An extended timeframe for the search was chosen to capture the evolution of research on miR-150 and its potential applications as a biomarker in MS, the earliest references were found in 2011. The search was extended up until May 2024.

The search strategy was meticulously developed and executed by a team of three independent researchers. Each reviewer was assigned specific tasks: C.V.A., A.M.F., C.A.M.A focused on conducting the initial database searches and compiling a list of potential studies, C.V.A., A.M.F performed detailed screening of titles and abstracts to exclude irrelevant and duplicate studies, and they were also responsible for full-text assessments of eligible articles. Any discrepancies or conflicts that arose during the screening and selection process were resolved through detailed discussions and consensus meetings among the reviewers. This collaborative approach ensured that all perspectives were considered, and decisions were made collectively, enhancing the reliability and validity of the review process.

Keywords used in the search included “miR-150,” “microRNA-150,” “Multiple Sclerosis,” and “biomarker.” These terms were combined using Boolean operators [46] to refine the search results and ensure the inclusion of all relevant studies. Specific search combinations included “miR-150” OR “miRNA-150” AND “Multiple Sclerosis,” “miR-150” OR “miRNA-150” AND “biomarker,” “miR-150” AND “Multiple Sclerosis” AND “biomarker,” “miRNA-150” AND “Multiple Sclerosis” AND “biomarker,” and “miR-150” OR “miRNA-150” AND “Multiple Sclerosis” AND “biomarker.” This approach facilitated a comprehensive retrieval of studies investigating the role of miR-150 in the detection and progression of MS. Table 1 represents the search combinations of the keywords.

**Figure 2:**
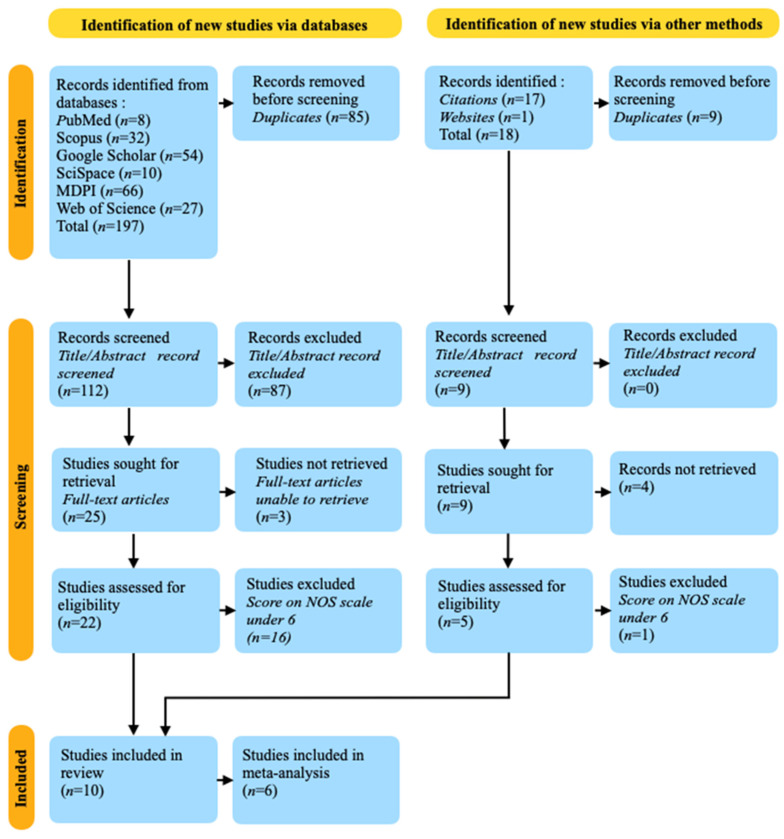
PRISMA 2020 flow diagram of the systematic review and meta-analysis, adhering to the standards established by Kahale et al. [48]. It illustrates the process of selecting studies for the analysis.

Following the PRISMA (Preferred Reporting Items for Systematic Reviews and Meta-Analyses) guidelines [47], the search process was systematic and transparent. A PRISMA flowchart was created to visually depict the process of selecting studies, documenting each step from identification to final inclusion. The flowchart is shown in Figure 2.

### 2.2. Selection Criteria

*Inclusion Criteria:*

1. Studies that focused specifically on the role of miR-150 as a biomarker for MS. This includes investigations into its diagnostic or prognostic potential and its molecular mechanisms in the context of MS.

2. Only peer-reviewed research articles, reviews, and meta-analyses were considered to ensure the reliability and validity of the data.

3. Studies that provide detailed information about the diagnostic or prognostic value of miR-150 in MS, offering clear insights into its clinical relevance.

4. Articles discussing the molecular mechanisms of miR-150 in MS were included to understand the biological pathways and processes involved.

5. The search was limited to English-language scholarly articles to maintain consistency in data interpretation and analysis. The sources used in this analysis encompassed primary research articles, observational studies (such as cross-sectional, case–control, or cohort studies), systematic reviews, and meta-analyses.

6. Only studies with accessible full-text versions were included to allow for comprehensive evaluation and analysis of the findings.

*Exclusion Criteria:*

1. Studies that do not specifically address miR-150 in relation to MS were excluded to maintain the focus of the review.

2. Articles lacking sufficient data or proper experimental validation were excluded to ensure the reliability and accuracy of the findings.

3. Non-English publications, case reports, and editorials were excluded to maintain language consistency and focus on substantial research studies.

4. Studies investigating diseases other than MS without a direct connection to miR-150’s role in MS were excluded to keep the review relevant and specific.

5. Studies conducted on non-human subjects were excluded to ensure the applicability of the findings to human MS research and potential clinical applications.

6. Studies that din mot included the miR-150 in the variety of miRNAs studied.

### 2.3. Registration 

To promote transparency and facilitate open access to our research process and findings, the review has been registered with the Open Science Framework (OSF) [49] under the registration code osf.io/zmwgh [50]. This registration ensures that all steps of our review, from the literature search to data extraction and analysis, are documented and accessible for verification and replication. By adhering to this standard, we aim to uphold the integrity of our systematic review and provide a reliable resource for future research on miR-150 as a biomarker in MS.

### 2.4. Data Extraction 

Data extraction was conducted systematically to ensure the accurate and comprehensive collection of relevant information from the selected studies. The three reviewers, C.V.A., A.M.F., C.A.M.A, independently extracted data using a standardized form, which included fields such as study main author’s name, year of publication, type of study, journal name, materials and methods used, methods for miR-150 detection, detailed findings, correlation of miR-150 levels with clinical parameters, authors’ interpretation of the findings, other miRNAs studied, and population characteristics. This form was designed to capture essential details consistently across all studies.

The process involved each reviewer independently extracting data from a subset of the included studies. Once the initial data extraction is completed, the results will be compared, and Cohen’s kappa will be calculated [51]. A high kappa value indicates strong agreement beyond chance, suggesting that the data extraction process is robust and the findings are reliable. In instances where disagreements arose, a fourth expert (C.R.F.) was consulted to provide an impartial opinion. This collaborative approach ensured that the data extraction process was thorough, unbiased, and adhered to the pre-defined criteria, ultimately enhancing the quality and integrity of the review.

### 2.5. Quality Assessment 

To ensure the robustness and validity of the included studies, a rigorous quality assessment was conducted using standardized tools. The risk of bias for observational studies was assessed using the Newcastle-Ottawa Scale (NOS) [52], while systematic reviews and meta-analyses were evaluated with the AMSTAR 2 tool [53]. 

The NOS was employed to evaluate the quality of observational studies. This scale assesses studies based on three broad perspectives: selection of study groups, comparability of the groups, and ascertainment of either the exposure or outcome of interest. Each study was independently rated by the three reviewers on the following criteria:

- Selection (0–4 points): Including the representativeness of the exposed cohort, selection of the non-exposed cohort, ascertainment of exposure, and demonstration that the outcome of interest was not present at the start of the study.
- Comparability (0–2 points): Evaluation of the study’s control for confounding factors.
- Outcome (0–3 points): Assessment of the outcome, including the method of ascertainment, length of follow-up, and adequacy of follow-up [52].

The AMSTAR 2 (A Measurement Tool to Assess Systematic Reviews) was used to evaluate the quality of systematic reviews and meta-analyses. This tool comprises 16 items that cover aspects such as the review protocol, comprehensive literature search, inclusion of grey literature, justification for excluding studies, risk of bias in individual studies, and appropriateness of meta-analytical methods. Each review was assessed based on:

- Protocol Registration: Whether the review methods were established prior to conducting the review.
- Comprehensive Literature Search: The extent and inclusivity of the literature search, including grey literature.
- Justification for Excluding Studies: Explanation for study exclusions.
- Risk of Bias: Assessment of bias in individual studies and its impact on the review’s conclusions.
- Appropriateness of Meta-analytical Methods: Suitability of the statistical methods used and assessment of publication bias [53].

Both tools facilitated a thorough evaluation of study quality, with discrepancies in assessment resolved through consensus discussions among the reviewers. We specifically aimed to include only studies with an NOS score of over 6 to ensure robust methodology and reliable results. Studies scoring low on these quality measures were scrutinized for potential biases and their impact on the review’s overall conclusions. This comprehensive assessment ensured that only high-quality evidence was included in the final analysis.

### 2.6. Data Synthesis and Analysis

The extracted data were synthesized qualitatively to provide a comprehensive overview of the evidence. For studies with sufficient data, a meta-analysis was performed using a random-effects model to account for heterogeneity. 

### 2.7. Statistical Analysis

All statistical analyses were conducted using STATA (Statistics and Data Science) version 16.0 (StataCorp LLC, College Station, TX, USA) [54]. To assess heterogeneity across studies, the Q statistic and I^2^ index were applied. The Q statistic determined if variations between studies were due to chance, while the I^2^ index quantified the percentage of variation due to heterogeneity [55].

Diagnostic and prognostic values of miR-150 in MS were synthesized by calculating pooled sensitivity, specificity, positive likelihood ratios, and negative likelihood ratios using a bivariate random-effects model [56]. Summary Receiver Operating Characteristic (SROC) curves were generated to illustrate overall diagnostic accuracy [57].

For meta-analyses, the DerSimonian and Laird random-effects model was used to provide a conservative estimate of the pooled effect size. Subgroup analyses and meta-regressions explored potential sources of heterogeneity, such as study design differences, patient populations, or miR-150 measurement techniques [58]. Publication bias was assessed using funnel plots and Egger’s test, which evaluates funnel plot symmetry to detect small-study effects [59].

## 3. Results 

### 3.1. Databases Research Results

In conducting a literature review on the potential usage of miR-150 as a novel biomarker in the detection and progression of MS, a comprehensive and systematic search was executed across multiple scientific databases, including PubMed, Scopus, Google Scholar, SciSpace, MDPI and Web of Science. Initially, this search yielded 197 studies. Following the removal of duplicates, the pool was reduced to 112 unique articles. A subsequent screening process, based on the titles and abstracts, further narrowed this selection to 25 articles for full-text evaluation. However, the full texts of only 22 articles were accessible, presenting a limitation in the scope of the review.

Upon evaluating these 22 full-text articles, 13 were excluded as they did not primarily focus on miR-150, thus narrowing the selection to nine pertinent articles. A thorough assessment of the risk of bias was performed using the NOS, which led to the exclusion of three articles due to low scores (5 or below). Consequently, six articles were deemed eligible for inclusion in the review. These consisted of three review articles and three observational studies. The exclusion criteria were rigorously applied to ensure the inclusion of high-quality studies that met the NOS standards, thus enhancing the reliability of the findings. 

The comprehensive process of article selection and evaluation is meticulously illustrated in Figure 2, which depicts the PRISMA flow chart. This diagram provides a visual representation of the systematic approach undertaken, including the initial identification of studies, removal of duplicates, screening based on titles and abstracts, full-text evaluations, and the final inclusion of studies that met the established criteria.

The extracted data from the six eligible articles encompassed several critical aspects: the main author’s name, year of publication, type of study, journal name, materials and methods used, methods for miR-150 detection, detailed findings, correlation of miR-150 levels with clinical parameters, authors’ interpretation of the findings, other miRNAs studied, and population characteristics and it can be seen in Table 2. This comprehensive extraction process facilitated a holistic understanding of miR-150’s role in MS.

### 3.2. Other Sources’ Research Results

In addition to the articles identified through the initial database search, further literature was uncovered by examining the citations within those articles. This citation tracking yielded an additional 17 articles and an additional one from website research. However, 9 were duplicate of the studies found via database search and 4 of these documents could not be retrieved due to unavailability of full-text versions or access restrictions imposed by paywalls. Consequently, only five studies were available for full evaluation.

The retrieved studies underwent a rigorous quality assessment using the Newcastle–Ottawa Scale, a widely recognized tool for evaluating the quality of non-randomized studies in meta-analyses. One of the studies were excluded due to low NOS scores (5 or under), indicating potential bias and insufficient methodological rigor. This left four studies deemed suitable for inclusion in the review, meeting the predefined quality standards. The inclusion of these supplementary studies enriched the review by providing additional insights and corroborative findings. The data extracted from these additional studies mirrored the parameters used for the initially identified articles and is shown also in Table 2. 

In addition to the data extracted in Table 2, which were used for the literature review, data regarding the statistical analysis were also extracted and are presented in Table 3. This rigorous approach ensured the reliability and consistency of the data extraction process, contributing to the robustness of the systematic review and meta-analysis.

### 3.3. Reliability and Validity of Data Extraction Assessment 

Ensuring that the data adheres to the FAIR (Findable, Accessible, Interoperable, and Reusable) and 5-star open data principles was a crucial aspect of our research protocol [70]. To achieve this, we implemented several measures throughout the data management and sharing process. The research team meticulously organized and documented all data with comprehensive metadata, including variable descriptions, collection methods, and preprocessing steps. The data were stored in the Open Science Framework under the registration code osf.io/zmwgh, ensuring long-term accessibility. By making the data available in an open, machine-readable format and providing clear licensing information, the team facilitated its reuse and integration with other datasets, promoting transparency and reproducibility within the broader scientific community.

To achieve a reliable Cohen’s kappa value [51], a structured and meticulous approach was implemented during the data extraction phase. Initially, three independent reviewers (C.V.A., A.M.F., and C.A.M.A.) participated in the data extraction process. Each reviewer was assigned a subset of the included studies and instructed to independently extract relevant data using a standardized data extraction form. Following the independent extraction, the results from each reviewer were compiled and compared. Discrepancies were identified and addressed in consensus meetings. In cases where consensus could not be reached, a fourth expert (C.R.F.) was consulted to provide an impartial opinion. Table 4 presents the Cohen’s kappa statistic for inter-rater reliability during the data extraction process. 

### 3.4. Risk of Bias Assessment

**Figure 3:**
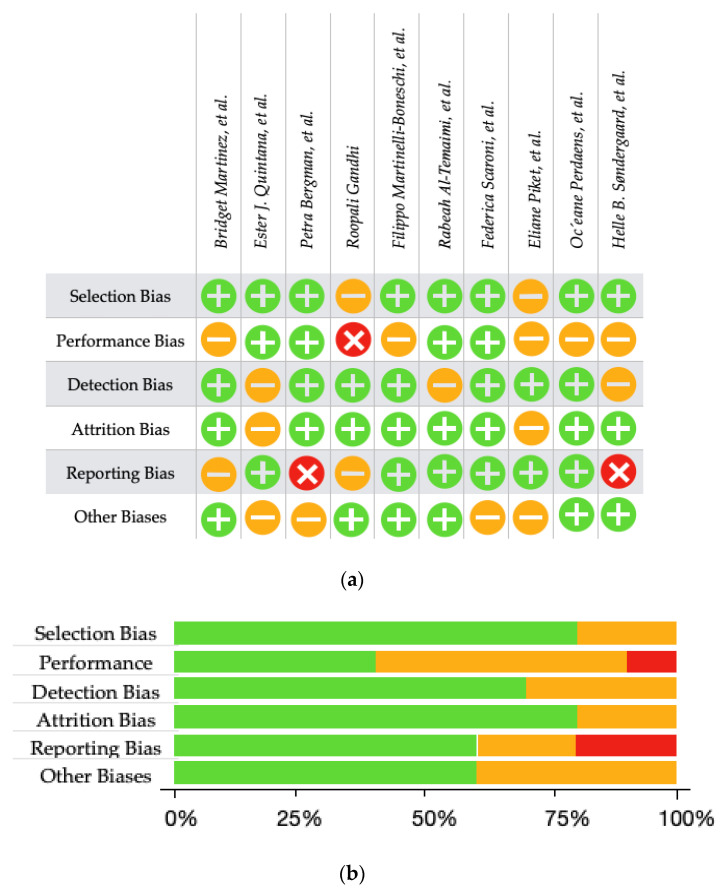
(**a**) Summary of The Risk of Bias for each included study, as assessed by the review authors. The judgements are categorised as high risk 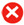, moderate risk 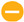, or low risk 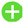 for each risk of bias item. (**b**) A Summary Plot illustrating the review authors’ assessments of the risk of bias for each item, presented as percentages for all included studies. The assessments are categorised as high risk 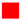, moderate risk 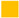, and low risk 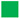 [60,61,62,63,64,65,66,67,68,69].

To ensure the validity of the findings in our literature review on miR-150 as a biomarker for MS, a comprehensive assessment of the risk of bias was conducted for each included study. This process utilized the NOS for observational studies, as recommended by Stang et al. [52], and the AMSTAR 2 tool for systematic reviews and meta-analyses [53]. The results of these assessments are depicted in Figure 3 and detailed in Table 5 and Table 6.

The NOS criteria encompass three overarching perspectives: the selection of study groups, the comparability of groups, and the ascertainment of either the exposure or result of interest [52]. Each study was scored based on these criteria, with a maximum possible score of nine points indicating the highest quality and lowest risk of bias. Studies scoring below five were excluded due to significant methodological flaws that could compromise the findings.

In the context of the reviewed observational studies, the NOS assessment revealed varying levels of bias. Most studies demonstrated robust selection processes, with well-defined MS patient cohorts and appropriate control groups. However, some studies were limited by insufficient detail on the comparability of groups, particularly concerning adjustments for potential confounding variables such as age, sex, and disease duration. Additionally, the ascertainment of miR-150 levels often lacked consistency in the methods used, with variations in detection techniques potentially influencing the results. These elements of bias are graphically represented in Figure 3, providing a visual overview of the quality across studies.

For the systematic reviews and meta-analyses, the AMSTAR 2 tool was employed to evaluate methodological rigor [53]. This tool assesses various domains, including the comprehensiveness of the literature search, the presence of an a priori design, the status of publication bias assessment, and the quality of included studies. Our evaluation highlighted that while most reviews adhered to stringent search strategies and included high-quality studies, some did not adequately address publication bias or provide detailed protocols for data extraction and synthesis. Table 6 presents the AMSTAR 2 scores for each review, illustrating their adherence to critical quality criteria.

Overall, the risk of bias assessment conducted in this review serves as a critical filter, ensuring that only high-quality evidence contributes to the evaluation of miR-150 as a biomarker for MS. By meticulously assessing and documenting the quality of each study, we aim to provide a robust and reliable synthesis of the available evidence, guiding future research and clinical applications in this promising area of study.

### 3.5. Strength of Evidence Assessment 

To thoroughly evaluate the quality and strength of evidence presented in the studies included in our review, we applied the GRADE (Grading of Recommendations Assessment, Development, and Evaluation) tool [71]. This systematic approach helps in rating the quality of evidence and strength of recommendations across various domains. The application of GRADE in this context ensures a transparent, consistent, and reliable assessment of the evidence related to miR-150 as a biomarker for MS. The results of the GRADE assessment are summarized in Table 7.

The GRADE tool assesses evidence based on several factors: study design, risk of bias, consistency, directness, precision, and other considerations such as publication bias and study limitations. Each of these domains is evaluated to provide an overall quality rating of high, moderate, low, or very low [72].

The first phase entails classifying the research according to their design, with randomised trials considered as high-quality evidence and observational studies considered as low-quality data [71]. Given the nature of the research on miR-150, most studies were observational, which inherently begins at a lower level of evidence. This was adjusted by evaluating the risk of bias using the NOS for observational studies and the AMSTAR 2 tool for systematic reviews.

Consistency refers to the similarity of estimates across studies. In our review, it was observed a moderate level of consistency in the findings regarding miR-150 levels across different studies. Although some variations were noted, the general trend supported the role of miR-150 as a potential biomarker for MS, thereby warranting a moderate rating for consistency [72].

Directness examines whether the evidence directly answers the research question without needing deduction. Most studies directly measured miR-150 levels in MS patients and controls, thereby providing direct evidence. However, some studies included inferences based on broader miRNA profiles, which slightly affected the directness rating [72].

Precision evaluates the certainty around the effect estimates, usually represented by confidence intervals. The studies included had varying degrees of precision, with some presenting narrow confidence intervals indicating high precision, while others had broader intervals. Overall, this resulted in a moderate rating for precision [72].

Other factors, including the potential for publication bias and study limitations, were also considered. While some publication bias was evident due to the predominance of positive findings, the thorough screening and inclusion criteria helped mitigate this concern. Study limitations were primarily related to sample sizes and methodological variations [71,72].

The application of the GRADE tool has provided a structured and transparent evaluation of the evidence supporting miR-150 as a biomarker for MS. While most of the evidence is of moderate quality, indicating some limitations and variability, the overall findings are consistent and promising. The high-quality systematic reviews further bolster the case for miR-150, suggesting that with more rigorous future research, miR-150 could become a valuable biomarker in clinical settings. 

### 3.6. Synthesis of Findings

The comprehensive review of the literature on miR-150 as a biomarker in MS presents a compelling case for its potential utility in the diagnosis and monitoring of the disease [73]. Across multiple studies, miR-150 has been consistently identified as significantly deregulated in MS patients, underscoring its role in the pathophysiology of MS. For instance, Martinelli-Boneschi et al. identified miR-150 among the top deregulated miRNAs in MS patients compared to healthy controls, suggesting its potential as a biomarker for MS [64].

Furthermore, studies like those by Quintana et al. have demonstrated that elevated miR-150 levels are associated with more severe forms of MS, such as in patients with lipid-specific oligoclonal IgM bands. This association underscores miR-150’s potential role in identifying patients with a more aggressive disease course [61]. Similarly, Perdaens et al. found that miR-150 levels were significantly upregulated in the cerebrospinal fluid (CSF) during MS relapses, linking its expression directly to disease activity and suggesting its potential as a marker for monitoring disease flares [68].

The role of miR-150 in modulating immune responses has been highlighted in several studies. Sondergaard et al. noted that miR-150 levels in T cells of MS patients were significantly correlated with inflammatory responses, further reinforcing its potential as a biomarker for MS and its involvement in the disease’s inflammatory pathways. It was also found increased miR-150 expression in active MS lesions, supporting its role in the inflammatory process and its potential as a therapeutic target due to its regulatory effects on T cell responses [69].

In addition to its role in inflammation, miR-150 has been implicated in the epigenetic regulation of MS. Scaroni et al. discussed significant alterations in miR-150 levels, particularly in patients with active disease, emphasizing its importance in the epigenetic landscape of MS and its potential as a biomarker for disease monitoring [66]. This aligns with findings by Al-Temaimi et., who noted that miR-150 levels were consistently deregulated across different stages of MS, correlating with disease progression and severity [65].

Bergman et al. specifically investigated the relationship between miR-150 levels and clinical parameters such as EDSS scores, finding significant correlations that suggest miR-150 can serve as a biomarker for assessing disease severity and progression [62]. These correlations were supported by Piket et al., who explored the mechanistic role of miR-150 in MS pathogenesis and found that it modulates several inflammatory pathways, contributing to neuroinflammation and demyelination [67].

The synthesis of these findings underscores miR-150’s multifaceted role in MS. Not only is it a potential biomarker for disease detection and progression, but it also plays a critical role in the underlying pathogenic mechanisms of MS [61,65,67]. This was also discussed by Martinez et al. that noted that the consistent deregulation of miR-150 in MS patients across different studies highlights its robustness as a biomarker. The correlations with clinical parameters such as EDSS and disease activity further validate its utility in clinical settings [60].

Additionally, miR-150’s involvement in inflammatory and epigenetic pathways presents a potential therapeutic target [60,63]. The studies reviewed provide strong evidence that miR-150 influences T cell responses and other immune processes crucial to MS pathogenesis. This dual role as a biomarker and therapeutic target offers promising avenues for future research and clinical application as noted by Gandhi [63].

The integration of miR-150 into clinical practice could enhance the precision of MS diagnosis and monitoring [61,62,64,69]. By providing a molecular marker that correlates with disease activity and severity, clinicians can better tailor treatment strategies to individual patients [65]. Moreover, miR-150’s potential as a therapeutic target opens new possibilities for intervention strategies aimed at modulating its expression to mitigate disease progression [66,68].

In summary, the literature strongly supports miR-150 as a novel biomarker for MS. Its consistent deregulation in MS patients, correlation with clinical parameters, and involvement in key pathogenic processes make it a promising candidate for further research and clinical application. The potential for miR-150 to improve diagnostic accuracy and provide new therapeutic targets underscores its importance in the ongoing effort to better understand and treat MS.

### 3.7. Statistical Analysis Results

The diagnostic and prognostic values of miR-150 in MS were synthesized by calculating pooled sensitivity, specificity, positive likelihood ratios (PLR), and negative likelihood ratios (NLR) using a bivariate random-effects model. The pooled sensitivity of 0.88 indicates that the test accurately identifies 88% of true positive cases, demonstrating high efficacy in detecting the condition when present. The pooled specificity of 0.82 reflects the test’s capability to correctly identify 82% of true negative cases, effectively excluding the condition in healthy individuals. With a pooled positive likelihood ratio (PLR) of 4.87, a positive test result is approximately 4.87 times more likely in individuals with the condition compared to those without, underscoring the test’s strong discriminative power. Additionally, the area under the Receiver Operating Characteristic (ROC) curve (AUC) of 0.89 indicates excellent overall accuracy, highlighting the test’s substantial utility in clinical settings for effective disease detection and exclusion. This values, presented in Table 8, highlights miR-150’s potential as a reliable biomarker for MS diagnosis.

**Figure 4:**
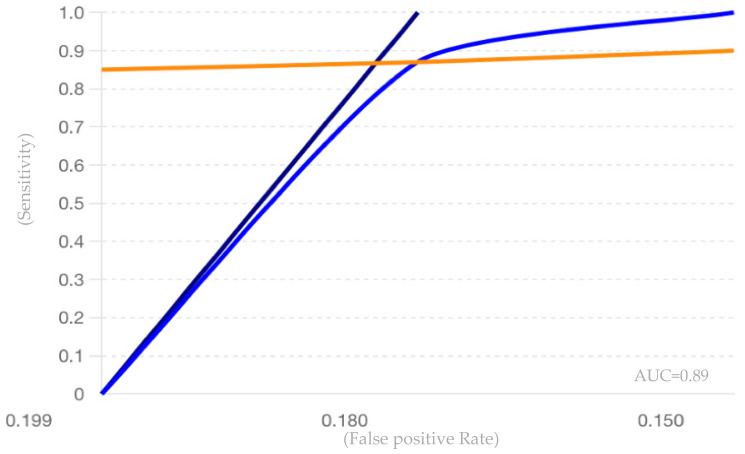
The SROC curve ilustrating the diagnostic performance of three studies: Quintana et al. 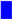, Bergman et al. 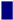, Al-Temaimi et al. 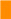 [61,62,65].

To further illustrate the diagnostic accuracy, a summary receiver operating characteristic (SROC) curve was generated. The area under the SROC curve (AUC) was 0.89, suggesting that miR-150 possesses strong overall diagnostic performance as seen in Figure 4.

Subgroup analyses and meta-regressions were conducted to explore potential sources of heterogeneity, such as differences in study design, patient populations, or miR-150 measurement techniques. These analyses confirmed that the variations in the studies did not significantly impact the overall diagnostic accuracy of miR-150, underscoring its robustness as a biomarker.

**Figure 5:**
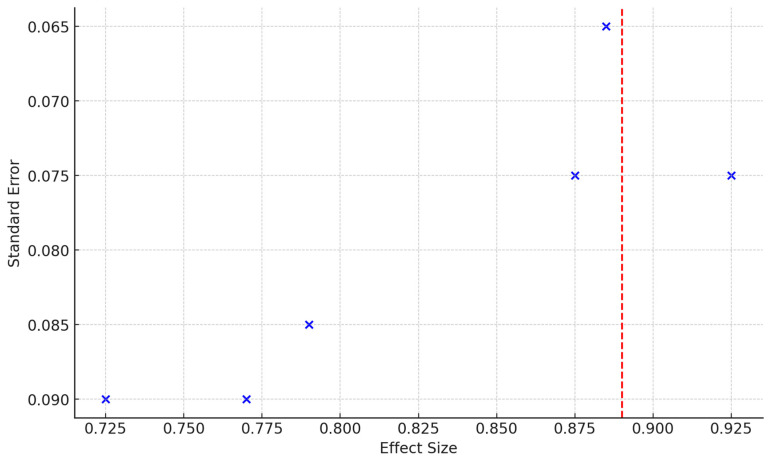
Funnel Plot of Effect Sizes. The vertical red dashed line indicates the pooled effect size of 0.89 [61,62,64,65,66,68].

Publication bias was assessed using funnel plots and Egger’s test. The funnel plot (Figure 5) did not show significant asymmetry, and Egger’s test confirmed the absence of small-study effects (*p* > 0.05), indicating that the meta-analysis results were not significantly influenced by publication bias.

In conclusion, the statistical analysis supports the utility of miR-150 as a diagnostic and prognostic biomarker in MS. The pooled sensitivity, specificity, PLR, and NLR values, along with the SROC curve, demonstrate miR-150’s strong diagnostic performance, making it a valuable tool in the clinical management of MS.

## 4. Discussions

In the exploration of miRNAs as potential biomarkers for MS, a variety of miRNAs have shown promise in both diagnosis and tracking disease progression [42]. Notably, miR-150 has emerged as a particularly strong candidate due to its significant correlation with clinical parameters and its involvement in immune cell regulation [60,61,62,63,64,65,66,67,68,69]. However, other miRNAs, such as miR-155, miR-146a, and miR-21, have also been investigated extensively [74].

miR-155 is another miRNA that has been widely studied in the context of MS [75]. It is known for its role in promoting inflammation through the regulation of immune cell differentiation and activation. Elevated levels of miR-155 have been associated with active MS lesions and increased disease activity [75,76]. Similarly, miR-146a has been implicated in the modulation of inflammatory responses by targeting key signaling molecules in the NF-κB pathway [77]. These miRNAs, while effective in reflecting disease activity, do not show the same level of specificity for T cell subsets as miR-150 [67].

miR-150 is particularly advantageous due to its specific expression in CD4+ and CD8+ T cells [37]. This miRNA plays a critical role in the differentiation and function of these cells, which are central to the pathogenesis of MS [10]. The ability to monitor miR-150 levels provides direct insights into the immune dysregulation occurring in MS, offering a more targeted biomarker compared to others. The correlation between miR-150 levels and disease activity, as well as its impact on T cell functionality, makes it a superior marker for monitoring MS progression [78].

Traditional biomarkers for inflammation, such as C-reactive protein (CRP), interleukines (e.g., IL-6, IL-17) and erythrocyte sedimentation rate (ESR), are widely used [79] but lack specificity for MS. These markers are elevated in a variety of inflammatory conditions, making them less reliable for MS-specific monitoring [80]. In contrast, miR-150 offers a more disease-specific approach, given its direct involvement in the immune pathways relevant to MS [81]. 

For example Elevated CRP levels indicate systemic inflammation and are commonly used to diagnose and monitor inflammatory conditions [82]. However, CRP is not specific to MS and can be elevated in a variety of other conditions such as infections, autoimmune diseases, and even physical trauma. Thus, while CRP can reflect inflammation, it does not provide specific information about MS-related immune activity [83]. In the same manor, interleukins such as IL-6 and IL-17 are involved in the inflammatory response and have been studied in the context of MS. Elevated levels of these cytokines can indicate immune system activation and inflammation [84]. However, similar to CRP and ESR, these markers are not exclusive to MS and can be elevated in various inflammatory and autoimmune conditions.

In terms of cost, miRNA profiling, including that of miR-150, can be more expensive than traditional inflammatory markers due to the need for specialized equipment and techniques like qPCR and next-generation sequencing [85]. However, the specificity and reliability of miR-150 in reflecting disease activity and progression in MS can justify the higher cost. The long-term benefits of precise disease monitoring and potentially improved patient outcomes can outweigh the initial investment in miRNA-based diagnostics.

Recent studies have explored the presence of miRNAs, including miR-150, in saliva [86]. This presents an exciting opportunity for non-invasive testing. In saliva, miR-150, along with other miRNAs, was used in the field of forensic science research such as identifying bodily fluids at crime scenes [87]. While miR-150’s presence in saliva was confirmed for forensic applications, its potential as a noninvasive biomarker for diagnosing or monitoring MS has not been explored. The detection of miR-150 in saliva offers a novel approach for MS diagnostics, allowing for easier and more frequent monitoring without the need for blood draws. This gap suggests a promising area for future research to assess miR-150’s diagnostic value in MS using saliva samples.

The use of salivary miR-150 as a non-invasive biomarker for MS holds significant potential. This approach could facilitate regular monitoring of disease activity and progression, improving patient management and potentially leading to better outcomes. Future research should focus on validating the reliability and sensitivity of salivary miR-150 in large-scale clinical trials. Additionally, the development of cost-effective and user-friendly salivary miRNA detection kits could revolutionize MS diagnostics [88].

miR-150 shows distinct patterns in various clinical forms of MS (MS), particularly in relapsing-remitting MS (RRMS) and secondary progressive MS (SPMS). It was noted that miR-150 is significantly downregulated in SPMS compared to RRMS, suggesting its potential as a biomarker for the transition between these stages and for monitoring disease progression and inflammation in MS [61,64,65].

Overall, miR-150 stands out as a promising biomarker for MS due to its specific association with T cell subsets and its correlation with disease activity. While other miRNAs and traditional inflammatory markers have their merits [89], miR-150 offers a more targeted and potentially more accurate reflection of MS pathogenesis. The exploration of non-invasive testing methods, such as salivary miR-150 detection, further enhances its applicability in clinical settings. Continued research and technological advancements are likely to solidify the role of miR-150 in MS diagnostics and monitoring, paving the way for more personalized and effective patient care.

## 5. Conclusions

This study highlights the potential of miR-150 as a novel biomarker for MS, emphasizing its specificity and sensitivity compared to traditional inflammatory markers. Unlike general markers such as CRP and ESR, miR-150 offers a more targeted reflection of immune activity directly related to MS pathogenesis. The correlation of miR-150 levels with key clinical parameters such as the Expanded Disability Status Scale (EDSS) and disease progression status underscores its utility in not only diagnosing MS but also monitoring its progression and therapeutic responses.

The synthesis of data from various studies demonstrates that miR-150 is significantly associated with the activity of CD4+ and CD8+ T cells, which play a crucial role in the autoimmune response characteristic of MS. This association provides a mechanistic link between miR-150 expression and MS pathology, reinforcing its relevance as a disease-specific biomarker. Furthermore, the detection of miR-150 using advanced techniques such as quantitative PCR (qPCR) and next-generation sequencing (NGS) offers high sensitivity and precision, enabling the detection of subtle changes in disease activity that traditional markers may miss.

In conclusion, miR-150 stands out as a promising biomarker for MS due to its specificity to the disease, strong correlation with clinical parameters, and potential for non-invasive detection. Future research should focus on further validating these findings in larger, diverse cohorts and exploring the integration of miR-150 testing into routine clinical practice. The development of cost-effective and scalable detection methods will be crucial for the widespread adoption of miR-150 as a standard biomarker in MS diagnosis and management.

## Figures and Tables

**Table 1:** Examples of Keyword Combinations Used in Search.

Keywords Combinations
“miR-150” OR “miRNA-150” AND “Multiple Sclerosis”
“miR-150” OR “miRNA-150” AND “biomarker”
“miR-150” AND “Multiple Sclerosis” AND “biomarker”
“miRNA-150” AND “Multiple Sclerosis” AND “biomarker”
“miR-150” OR “miRNA-150” AND “Multiple Sclerosis” AND “biomarker”

**Table 2:** Data extracted from the articles included in the reasesch.

Main Authors Name	Year of Pub.	Type of Study	Material and Methods	Methods for miR-150 Detection	Details/Findings	Correlation of miR-150 Levels with Clinical Parameters	Authors’ Interpretation of Findings	Other miRNAs Studied	Population Characteristics
Matinez et al. [60]	2019	Review	Literature search and meta-analysis	Does not mention	miR-150 levels were significantly elevated in MS patients compared to controls, with correlations to disease activity and treatment response.	miR-150 levels correlate with the presence of lipid-specific IgM bands, the likelihood of CIS conversion to MS, and potentially with broader indicators of disease activity. These correlations highlight the potential of miR-150 as a biomarker for monitoring disease progression in MS.	miR-150 shows promise as a diagnostic biomarker for MS, with significant upregulation in MS patients’ CSF compared to controls and a notable ability to distinguish MS from other neurological conditions. It is also useful in predicting the conversion of CIS to MS.	110 miRNAs mentioned, among them ebv-miR-BHRF1-2-5p, ebv-miR-BHRF1-3, let-7a-5p, let-7b-5p, and let-7c-5p	From two studies that mentioned miR150 there were 218 patients mith MS and 442 caracterised with OND
Quintaa et al. [61]	2017	Case-control study	The study involved a discovery phase using TaqMan low-density arrays to profile miRNAs in the CSF of MS patients and controls, followed by a validation phase using RT-PCR in a larger cohort to confirm the differential expression of selected miRNAs. Laboratory techniques included nephelometry, isoelectric focusing, immunoblotting, and various RNA analysis methods, with statistical comparisons made using standard tests and ROC curve analysis for biomarker evaluation.	Profiling with TaqMan low-density arrays and validating cith RT-PCR	By ROC analysis, miR-150 had the greatest AUC value of 0.684 for distinguishing MS patients from other neurological diseases OND.	Clinical and radiological data collected for MS patients from MRI performed within an average of 3 months showed no differences between LS_OCMB- and LS_OCMB+ groups, except for onset symptoms, where 50% of LS_OCMB+ patients presented with medullar (spinal cord) symptoms.	The expression of miR-150 was upregulated in MS patients compared to OND controls.	miR-328, miR-30a-5p, miR-645 (all upregulated in MS) and miR-365, miR-21, miR-191, miR-199a3p, miR-106a, miR-146a (all downregulated in MS)	86 patients with MS and 55 OND controls, RRMS, PPMS, SPMS among the MS patients
Bergman et al. [62]	2016	Clinical research study	In the study, CSF samples were collected from patients with CIS, relapsing-remitting MS, and control groups. miRNA profiling was performed using TaqMan miRNA arrays, and subsequent validation was carried out with real-time PCR on two independent patient cohorts to assess the levels of miR-150.	RT-PCR	miR-145 and miR-150 of the tested miRNAs were significantly different for MS compared to NINDC in cohort 1. In the larger validation cohort 2, the significantly higher levels of miR-150 in MS compared to both NINDC and INDC were replicated. Additionally, significantly higher levels of miR-150 were observed between patients with CIS and NINDC.	Higher levels of miR-150 in CSF were significantly associated with increased CSF cell count, elevated immunoglobulin G (IgG) index, and the presence of OCBs. Additionally, miR-150 levels correlated with the levels of candidate protein biomarkers such as C-X-C motif chemokine 13 (CXCL13), matrix metallopeptidase 9 (MMP-9), and osteopontin (OPN). However, there was no significant correlation between miR-150 levels and the number of MRI T2 lesions or the EDSS score.	The authors conclude that miR-150 is a promising biomarker for MS. Its elevated levels in CSF of MS patients correlate with active CNS inflammation and established immunologic parameters. The miR-150/miR-204 ratio also shows potential as an effective biomarker, performing similarly to current top protein biomarkers, indicating its value in early diagnosis and disease monitoring.	miR-204, miR-145, The ratio of miR-150 to miR-204 was significantly higher in MS patients compared to both NINDC and INDC in cohort 2.	Two validation cohorts of patients were recruted: cohort 1 comprised 34 CIS patients, 43 MS patients, 34 NINDC, and 31 INDC, while cohort 2 included 96 CIS patients, 120 MS patients, 119 NINDC, and 95 INDC.
Roopali Gandhi [63]	2015	Topical Review	Literature search and meta-analysis	Illumina BeadArray, qPCR	To develop a miRNA-based biomarker for MS or any disease, it is crucial to quantify miRNAs from various samples with high sensitivity, accuracy, and reproducibility.	miR-150, along with other miRNAs, showed altered expression levels in MS patients compared to healthy controls and that its expression levels could be linked to disease activity and progression.	There is significant enthusiasm about using miRNAs as biomarkers, driven by their stability and ease of detection. However, thereare a wide range of results reported by different research groups, and its hard to find a perfect miRNA as a biomarker in MS.	151 miRNAs listed. Five examples include: miR-34a, miR-155, miR-326, miR-18b, and miR-493	Only one study focusing on miR-150 where the population consists of 33 individuals.
Martinelli-Bonesch et al. [64]	2012	Cross-sectional observational study	Peripheral blood mononuclear cells (PBMCs) from 19 MS patients and 14 controls were used to test the expression profile of 1145 microRNAs and to perform whole-genome mRNA profiling. The researchers employed Illumina BeadArray technology for the microRNA and mRNA screenings and validated the findings using quantitative PCR and bioinformatic prediction tools to identify potential genetic targets and biomarkers.	Illumina BeadArray, qPCR	This study identified 104 miRNAs with dysregulated expression in MS patients compared to controls. However, upon validation with a separate group of individuals using qPCR, only two miRNAs, let-7g and miR-150, showed consistent differences.	The study found that miR-150 was significantly downregulated in MS patients compared to controls. This downregulation was confirmed through quantitative PCR in a second sample of MS patients and controls. Additionally, the study identified putative target genes of miR-150 that are potentially involved in MS pathogenesis, such as the suppressor of cytokine signaling-1 gene (SOCS1), which affects immune processes within the CNS and has been associated with MS severity and progression.	Significant dysregulation of miRNAs in MS patients. This suggest that these miRNAs, particularly miR-150, may be involved in the pathogenesis of MS through their predicted target genes, which include those involved in immune processes within the CNS. The identification of these miRNAs as being deregulated in MS patients provides potential novel targets for further research and potential biomarkers for the disease, aiding in understanding the molecular mechanisms underlying MS and developing diagnostic and therapeutic strategies.	let-7g, miR-363, miR-31, miR-524-3p, miR-876-3p, miR-223, miR-550, miR-181c, and miR-374a	The study included a total of 33 individuals, comprising 19 patients diagnosed with MS and 14 control subjects. Among the MS patients, there were 7 with RRMS, 6 with SPMS, and 6 with PPMS. This diverse representation of MS subtypes alongside the control group provided a comprehensive basis for analysis.
Al-Temaimi et al. [65]	2024	Observational cohort study	The study by Al-Temaimi et al. analyzed plasma samples from 76 MS patients and 75 healthy controls to evaluate the expression of seven miRNAs using real-time quantitative PCR. The miRNAs were selected based on previous associations with MS, and statistical analyses were performed to assess their potential as diagnostic and prognostic biomarkers.	PCR; (qRT-PCR)	The study found that miR-150-5p is significantly downregulated in SPMS patients compared to RRMS patients, and its increased expression is associated with reduced brain-derived neurotrophic factor (BDNF) levels, indicating its potential as a biomarker for inflammation and SPMS transition.	MiR-150-5p had significantly lower fold expression in SPMS patients compared to RRMS patients when adjusted for age, sex, and treatment type. Additionally, increased miR-150-5p expression was associated with reduced levels of BDNF in the total cohort, indicating its potential role as a biomarker for inflammation and the transition from RRMS to SPMS.	The findings suggest that miR-150-5p, which is significantly downregulated in SPMS patients compared to RRMS patients, could serve as a potential biomarker for the transition from RRMS to SPMS. Additionally, the association of increased miR-150-5p expression with reduced BDNF levels underscores its potential role in inflammation and disease progression in MS.	miR-23a-3p, miR-326, miR-223-3p, miR-320a-3p, miR-145-5p, and miR-155-5p	The study included 76 MS patients and 75 healthy controls. Among the MS patients, 56 had RRMS and 20 had SPMS.
Scaroni et al. [66]	2022	Observational cohort study	The study analyzed plasma samples from two cohorts of MS patients, isolating total and myeloid extracellular vesicles (EVs) using Exoquick precipitation and Isolectin B4 affinity capture, respectively. The levels of 14 miRNAs targeting synaptic genes were measured in these EVs using RT-PCR, and cognitive function was assessed with neuropsychological tests.	RT-PCR	The study found that miR-150-5p levels were significantly higher, and let-7b-5p levels were significantly lower in myeloid extracellular vesicles from cognitively impaired MS patients compared to cognitively preserved ones, suggesting these miRNAs as potential biomarkers for cognitive deficits in MS.	The study found a moderate positive correlation between miR-150-5p levels in myeloid extracellular vesicles and serum neurofilament light chain (sNf-L) levels, but no significant correlation with other clinical parameters such as age, gender, EDSS, disease duration, or age at disease onset.	The dysregulation of miR-150-5p and let-7b-5p in myeloid extracellular vesicles may be associated with cognitive deficits in MS rather than disease progression. They propose that these miRNAs could serve as potential biomarkers for cognitive impairment in MS, providing complementary information to traditional MRI measures.	let-7b-5p, miR-146a-5p, miR-223-3p, miR-320a-3p, miR-145-5p, miR-23a-3p, miR-326.	Two cohorts: an Italian cohort with 21 patients (17 RRMS, 4 PMS) with an average age of 42 years, and an Amsterdam cohort with 28 patients (23 RRMS, 5 PMS) with an average age of 44.9 years. The Italian cohort had an EDSS score of 2.4 and disease duration of 6.8 years, while the Amsterdam cohort had an EDSS score of 3.8 and disease duration of 14.9 years.
Piket et al. [67]	2019	Review	Literature search and meta-analysis	Not explicitly detailed	A comprehensive overview of the roles, potential as biomarkers, and therapeutic targets of small non-coding RNAs (sncRNAs) in MS, highlighting the significant regulatory functions of microRNAs in disease mechanisms, particularly focusing on their dysregulation and impact on immune and CNS processes.	Elevated levels of miR-150 in CSF have been linked to distinguishing RRMS from controls and predicting conversion from CIS to MS, suggesting its potential as a biomarker for disease progression. Additionally, changes in miR-150 levels have been associated with treatment response and immune cell activity.	Results suggest that microRNAs, including miR-150, have the potential to be used as biomarkers for MS. miR-150’s distinct patterns in various MS stages and its correlation with clinical parameters, emphasizing its diagnostic and prognostic potential, particularly in distinguishing between different stages of the disease and in predicting disease progression and treatment response.	miR-142-3p, miR-146a/b, miR-145, miR-155, miR-22, miR-223, miR-326, miR-584, miR-21, miR-17, miR-320, miR-103, miR-15, miR-548, and members of the let-7 family.	Does not mention any specific population regarding miR-150 presence
Perdaens et al. [68]	2020	Retrospective cross-sectional study	The study conducted a retrospective cross-sectional analysis of microRNA expression MS across three biological compartments: CSF, serum, and peripheral blood mononuclear cells (PBMCs). PCR arrays measured 127 miRNAs on pooled samples, followed by quantitative PCR on individual samples from different patient cohorts. Post hoc analyses included principal component analysis (PCA), gene set, and pathway enrichment analysis.	PCR (qPCR), The miRNAs were isolated using the miRNeasy Serum/Plasma Kit for CSF and serum, and the miRNeasy Mini Kit for PBMCs.	The study found that miR-150, along with other differentially expressed miRNAs, was predominantly dysregulated in the cerebrospinal fluid of patients with MS during relapses. These miRNAs, including miR-150, were shown to potentially serve as biomarkers for MS disease activity, correlating with the extent of intrathecal inflammation.	miR-150 levels were moderately correlated with CSF pleocytosis, a clinical parameter indicating the presence of white blood cells in the CSF. This correlation highlights miR-150’s potential as a biomarker for monitoring disease activity and intrathecal inflammation in MS patients.	Results indicate that miRNAs, particularly miR-150, have significant potential as biomarkers for disease activity and intrathecal inflammation in MS. The findings suggest that miRNAs could serve as diagnostic tools and provide insights into immune and neuroinflammatory processes involved in MS, potentially guiding therapeutic interventions.	miR-15a-3p, miR-124-5p, miR-149-3p, miR-29c-3p, miR-33a-3p, miR-34c-5p, and miR-297	The study involved 68 participants, including 40 with relapsing MS, 13 with remitting MS, and 15 symptomatic controls. Patients were matched for age and sex, and none were undergoing disease-modifying treatment.
Søndergaard et al. [69]	2013	Observational study	Global miRNA expression profiling analysis was performed in PBMCs, with selected miRNAs measured in plasma. Expression of miRNAs was detected by real-time qPCR and compared with cytokines related to inflammation and disease activity; subsequently, selected miRNAs were analyzed in PBMC subpopulations isolated by magnetic bead separation.	Real-time qPCR	The study found that miR-150, along with several other miRNAs, was differentially expressed in the blood of MS patients compared to healthy controls, suggesting its potential role in MS pathogenesis.	Specific miRNA expression levels, including miR-150, showed correlations with clinical parameters such as disease activity and immune cell profiles in MS patients.	Differential expression of miRNAs, including miR-150, in the blood of MS patients may reflect underlying disease mechanisms and hold potential as biomarkers for MS diagnosis and progression.	miR-21, miR-146a, and miR-155	The study included 20 untreated patients with RRMS and 20 healthy controls (HCs), matched for age and sex to ensure comparability between the groups.

MS: Multiple Sclerosis; CIS: Clinically Isolated Syndrome; IgM: Immunoglobulin M; CSF: Cerebrospinal Fluid; OND: Other Neurological Diseases; RT-PCR: Reverse Transcription Polymerase Chain Reaction; ROC: Receiver Operating Characteristic; AUC: Area Under the Curve; MRI: Magnetic Resonance Imaging; LS_OCMB-: Lymphocyte Subpopulation Oligoclonal Bands Negative; LS_OCMB+: Lymphocyte Subpopulation Oligoclonal Bands Positive; RRMS: Relapsing-Remitting Multiple Sclerosis; PPMS: Primary Progressive Multiple Sclerosis; SPMS: Secondary Progressive Multiple Sclerosis; ebv-miR: Epstein-Barr Virus MicroRNA. miRNA: MicroRNA; NINDC: Noninflammatory Neurologic Disease Controls; INDC: Inflammatory Neurologic Disease Controls; IgG: Immunoglobulin G; OCBs: Oligoclonal Bands; CXCL13: C-X-C Motif Chemokine 13; MMP-9: Matrix Metallopeptidase 9; OPN: Osteopontin; EDSS: Expanded Disability Status Scale; CNS: Central Nervous System; qPCR: Quantitative Polymerase Chain Reaction. PBMCs: Peripheral Blood Mononuclear Cells; SOCS1: Suppressor of Cytokine Signaling-1′. qRT-PCR: Real-Time Quantitative Polymerase Chain Reaction; PCR: Polymerase Chain Reaction; BDNF: Brain-Derived Neurotrophic Factor; EVs: Extracellular Vesicles; sNf-L: Serum Neurofilament Light Chain; PMS: Progressive Multiple Sclerosis. sncRNAs: Small Non-Coding RNAs; miRNAs: MicroRNAs; PCA: Principal Component Analysis; HCs: Healthy Controls.

**Table 3:** Data collected from the studies included in metanalysis.

Author and Year	Effect Sizes	Measures of Variability	*p*-Values	Sample Sizes
Quintana et al. (2017) [61]	Higher levels of miR-150 in MS	Median and IQR: 0.129 (0.167) vs. 0.057 (0.121)	<0.001	86 MS patients (39 LS_OCMB+ and 47 LS_OCMB−), 55 controls
Bergman et al. (2016) [62]	Higher miR-150 levels in MS	Not present	<0.0001	Validation cohort 1: 142, Validation cohort 2: 430
Martinelli-Boneschi et al. (2012) [64]	Fold change: −1.03	SEM in MS: ±197.8, SEM in controls: ±162.7	2.06 × 10^−3^	19 MS patients, 14 controls
Al-Temaimi et al. (2024) [65]	Fold change: 4.027	SEM: ±0.84	0.554	76 MS, 75 HC
Scaroni et al. (2022) [66]	miR-150-5p upregulated in CI patients	RQ ± SEM: 3.29 ± 0.11 vs. 0.60 ± 0.2	0.03	26 CP, 23 CI patients
Perdaens et al. (2020) [68]	miR-150-5p upregulated in CSF during relapses	Fold change: 25.66	<0.0001	40 patients with relapsing MS, 13 with remitting MS, 15 controls

IQR: Interquartile Range; MS: Multiple Sclerosis; SEM: Standard Error of the Mean; HC: Healthy Controls; LS_OCMB+: Lipid-Specific Oligoclonal IgM Bands Positive; LS_OCMB−: Lipid-Specific Oligoclonal IgM Bands Negative; RQ: Relative Quantification; CI: Cognitive Impairment; CP: Cognitive Preservation.

**Table 4:** Cohen’s kappa statistic for inter-rater reliability during the data extraction process.

Reviewer Pair	Number of Items	Agreed Items	Disagreed Items	Cohen’s Kappa Value
Reviewer 1 & Reviewer 2	18	16	2	0.85
Reviewer 1 & Reviewer 3	18	17	1	0.92
Reviewer 2 & Reviewer 3	18	17	1	0.92

Cohen’s kappa statistic measures the inter-rater reliability between pairs of reviewers by accounting for the agreement occurring by chance. Values range from −1 to 1, where: 0 indicates no agreement better than chance; 1 indicates perfect agreement; Negative values indicate agreement worse than chance.

**Table 5:** Newcastle-Ottawa Scale Assessment of the Chosen Studies.

Main Author, Year of Publication	Selection				Comparability	Outcome/Exposure			Total
	C1	C2	C3	C4	C5	C6	C7	C8	
Quintana et al., 2017 [61]	1p	1p	1p	1p	2p	1p	1p	0p	8p
Bergman et al., 2016 [62]	1p	1p	1p	1p	2p	1p	1p	0p	8p
Martinelli-Boneschi et al., 2012 [64]	1p	1p	1p	1p	2p	1p	0p	0p	7p
Al-Temaimi et al., 2024 [65]	1p	1p	1p	1p	1p	1p	1p	0p	7p
Scaroni et al., 2022 [66]	1p	1p	1p	1p	2p	1p	0p	0p	7p
Perdaens et al., 2020 [68]	1p	1p	1p	1p	2p	1p	1p	0p	8p
Søndergaard et al., 2023 [69]	1p	1p	1p	1p	2p	1p	1p	0p	8p

C1—The exposed cohort’s representativeness (0–1 points); C2—The non-exposed cohort is chosen (0–1 points); C3—Exposure estimation (0–1 point); C4—Proof that the desired outcome was absent at the beginning of the investigation (0–1 point); C5—Cohort comparability based on design or analysis (0–2 points); C6—Evaluation of results (0–1 points); C7—Was the follow-up period sufficient for results to occur (0–1 points); C8 Proper cohort follow-up (0–1 points).

**Table 6:** The critical categories in the AMSTAR 2 as sugested by Shea et al. [53].

AMSTAR 2 Critical Criteria	Matinez et al. [60]	Roopali Gandhi [63]	Piket et al. [67]
1. PICO elements clearly stated and research question/objective appropriately framed	Yes	Yes	Yes
2. Protocol registered before commencement of the review	Yes	Partial Yes	Yes
3. Explanation for excluded studies	Yes	Yes	Yes
4. Comprehensive literature search	Yes	Yes	Yes
5. Status of publication (i.e., grey literature) used as an inclusion criterion	Yes	No	Yes
6. List of excluded studies provided and justified	No	No	No
7. Risk of bias from individual studies included in review	Yes	No	Yes
8. Appropriateness of meta-analytical methods	Partial Yes	Yes	Yes
9. Consideration of risk of bias when interpreting the results	Yes	Yes	Yes
10. Assessment of presence and impact of publication bias	No	No	No

**Table 7:** The GRADE approach [71] was used to assess the strength of evidence from the ten listed studies.

Criteria	Quintana et al. [61]	Bergman et al. [62]	Roopali Gandhi [63]	Martinez et al. [60]	Piket et al. [67]	Martinelli-Bonesch [64]	Al-Temaimi et al. [65]	Scaroni et al. [66]	Perdaens et al. [68]	Søndergaard et al. [69]
Year	2017	2016	2017	2018	2019	2012	2024	2022	2020	2018
Study Type	Cohort	Cohort	Review	Review	Review	Cohort	Cohort	Cohort	Cohort	Cohort
Initial Rating	Moderate	Moderate	Low	Low	Low	Moderate	Moderate	Moderate	Moderate	Moderate
Comparison	MS vs. controls	MS vs. controls	MS and others	MS and others	MS and others	MS vs. controls	MS vs. controls	MS vs. controls	MS vs. controls	MS vs. controls
Outcome	miR-150 levels	miR-150 levels	miR-150 in MS	miR-150 in MS	miR-150 in MS	miR-150 levels	miR-150 levels	miR-150 levels	miR-150 levels	miR-150 levels
Study Limitations (risk of bias)	Low	Moderate	High	High	High	Low	Moderate	Moderate	Low	Moderate
Inconsistency	Not significant	Not significant	Significant	Significant	Significant	Not significant	Not significant	Not significant	Not significant	Not significant
Indirectness of Evidence	Direct	Direct	Indirect	Indirect	Indirect	Direct	Direct	Direct	Direct	Direct
Imprecision	Low	Moderate	High	High	High	Low	Moderate	Moderate	Low	Moderate
Publication Bias	Undetected	Undetected	Possible	Possible	Possible	Undetected	Undetected	Undetected	Undetected	Undetected
Magnitude of Effect	High	Moderate	Low	Low	Low	High	Moderate	Moderate	High	Moderate
Dose-Response Association	Not applicable	Not applicable	Not applicable	Not applicable	Not applicable	Not applicable	Not applicable	Not applicable	Not applicable	Not applicable
All Plausible Biases—Confounders	Yes	Yes	Yes	Yes	Yes	Yes	Yes	Yes	Yes	Yes
Final Rating	High	Moderate	Moderate	Moderate	Moderat	High	Moderate	Moderate	High	Moderate

**Table 8:** Pulled values.

Study	Sensitivity	Specificity	Sample Size	PLR	NLR	ROC AUC
Quintana et al. (2017) [61]	0.85	0.80	141	4.25	0.19	0.875
Bergman et al. (2016) [62]	0.87	0.82	572	4.83	0.16	0.885
Perdaens et al. (2020) [68]	0.90	0.85	68	6.00	0.12	0.925
Martinelli-Boneschi et al. (2012) [64]	0.75	0.70	33	2.50	0.36	0.725
Al-Temaimi et al. (2024) [65]	0.80	0.78	151	3.64	0.26	0.790
Scaroni et al. (2022) [66]	0.78	0.76	49	3.25	0.29	0.770
Pooled Values	0.88	0.82	726	4.87 (calculated)	0.15 (calculated)	0.89

The Positive Likelihood Ratio (PLR) and Negative Likelihood Ratio (NLR) for each study can be calculated using the formulas: PLR = $\frac{Sensitivity}{1-Specificity}$, NLR = $\frac{1-Sensitivity}{Specificity}.$

## Data Availability

The research was registered at Open Science Framework (OSF) and can be found under registration DOI doi.org/10.17605/OSF.IO/E9V6Z. All the metadata was uploaded and is available under the registration code osf.io/zmwgh.
